# Five hundred million years of hunger: how animals evolved to survive essential amino acid scarcity

**DOI:** 10.1042/ETLS20253009

**Published:** 2025-12-24

**Authors:** Benjamin S. Pickard

**Affiliations:** 1 Strathclyde Institute of Pharmacy and Biomedical Sciences, University of Strathclyde, Glasgow, G4 0RE, U.K.

**Keywords:** amino acid metabolism, evolutionary biology, gene expression and regulation, homeostasis, obesity, protein synthesis

## Abstract

Proteins are the machinery for the processes of life. Each protein is made up of a defined combination of 20 building blocks, the amino acids. The animal kingdom is distinguished from most other forms of life by a half-billion-year-old choice to relinquish the synthesis of 9 of the 20 amino acids and instead rely on their dietary acquisition for protein synthesis. From that point onwards, animals entered into a permanent and obligatory hunt for these ‘essential amino Acids’ (EAAs).

This perspective states that this seemingly destructive event was, in fact, foundational for the animal kingdom. Hypotheses for its origins are discussed, including a newly observed bias in EAA codon nucleotide composition that may help economise their use in proteins during scarcity. Tight restrictions on the inclusion of EAAs in protein sequences would be expected, but a minority of proteins with extreme EAA compositions are found. It is hypothesised that such proteins act as sentinels of EAA shortage in the diet, prompting beneficial responses from the organism. The control of hunger behaviours and reproductive timing are two processes in which EAA-rich proteins may be important. The leptin pathway of hunger behaviour regulation and reproductive development, traditionally associated with bodily lipid homeostasis, may be sensitive to EAA levels through this sequence-based mechanism.

EAAs appear to have been a strong force in animal evolution. The biology emerging from their patterns of use in our proteins provides a direct link between nutritional state and specific biological processes – a coherent route to better dietary interventions in the future.

## Introduction

### The origins of essential amino acids: a counterintuitive decision

*A new policy requires that 45% of agricultural land area should be immediately repurposed for housing and industrial development, and the population sustained by means of an expanded international food supply chain.*

This hypothetical directive would rightfully be derided as impulsive and short-sighted. And yet, sometime between 500 and 550 million years ago, this is, in essence, the path that the animal kingdom chose: the biosynthetic pathways that synthesised 9 of the 20 proteogenic amino acids were abandoned as their constituent enzyme-encoding genes escaped selective pressure and drifted into mutation and loss. At each mutational step, an evolutionary cost–benefit calculation must have determined the relative risk of amino acid starvation against the biosynthetic energy savings gained by outsourcing supply. For nine amino acids (at that time), every calculation favoured pathway loss, suggesting that dietary supply must have been plentiful enough to compensate for lack of synthesis. However, the decision to take one step up the food pyramid meant that animals would be forever hungry for what we now term the essential amino acids (EAAs). This amino acid classification was first defined 75 years ago by trialling restrictive diets on rodents and determining which amino acids were ‘indispensable’ for survival [1,2]. Of the other amino acids, a further six, the conditionally essential amino acids (CEAAs), can still be synthesised but, in times of stress, proliferation, development, or illness, these, too, require dietary supplementation. Only the five non-essential amino acids (NEAAs) are adequately provided through internal biosynthesis.

In the millennia since animals lost the ability to synthesise EAAs, the supercontinent Pangea formed and broke apart, the average surface temperature on Earth oscillated between 11°C and 36°C [3], and five major extinction events have radically shuffled and re-shuffled the planet’s portfolio of life. There were clearly no guarantees that the environment that once sustained the animal kingdom’s choice to drop EAA synthesis would continue to do so. Despite this uncertainty, the plain observation is that animals have survived and thrived through this tumult. Indeed, perhaps it is no coincidence that the Cambrian explosion in animal diversity, complexity, and number occurred at very approximately the same time (539 mya) as the nine amino acids became essential. Many strong candidates, such as increased atmospheric oxygen and greater calcium availability, have been suggested as triggers for this burst of animal evolution [4]. Perhaps, the absolute dependence on eating other species (including other animals) to acquire EAAs should also join these Cambrian drivers of protective, exoskeletal calcified defences, varied methods of locomotion, a coelom for digestion and motility, and the rostral clustering of sensory, information processing, and buccal functions.

Nevertheless, it cannot be ignored that the presence of the word ‘essential’ in the nutritional amino acid classification system establishes a hard border between survival and death. This should prompt speculation that the acquisition of food and its allocation to proteins have been locked into half a billion years of fierce natural selection. In this perspective, two hypotheses are set out that speculate on the origin of the EAAs, and how they may have impacted the evolution of the animal proteome.

### The great genome deletion in animals – and others?

The EAAs are valine, isoleucine, leucine, lysine, histidine, methionine, phenylalanine, tryptophan, and threonine. Some lower animals also lack the ability to make a tenth amino acid, arginine. Before discussing the impacts of their shortage, it is necessary to ask, why these particular amino acids? The ‘Great Genome Deletion’ is the term coined to describe the ancient sweep of amino acid biosynthetic pathway loss that we see evidenced today in all modern multicellular animals (metazoans) [5]. However, amino acid essentiality has also been described in several unicellular eukaryotic phyla, including those containing species such as *Dictyostelium discoideum and Tetrahymena thermophila*. In each instance, it is the same nine (or ten) amino acids that have become essential [5,6], although *Dictyostelium* has also added serine to the list. The most straightforward explanation is that the animal kingdom and these other phyla can be traced back to a common ancestor that underwent the primary Great Genome Deletion event. However, some have suggested that the precise phylogenetic branchpoints of these phyla require multiple independent deletion events to have occurred. Exact repetition of deletion in distinct events is difficult to explain – the nine EAA biosynthetic pathways are metabolically disparate (except for the branched-chain amino acids, isoleucine, leucine, and valine; and the amino acids phenylalanine and tryptophan), so a simple pruning of one branch of the biochemical tree does not explain the exact repetition of loss [6]. One persuasive hypothesis is that these tend to exhibit hydrophobicity and are the most energetically expensive amino acids to synthesise [7], so their loss is just a top-slicing process. As a partial counter to this argument, tyrosine, cysteine, and alanine should also be essential if these criteria were the only explanation. Another hypothesis for independent emergence suggests that the spectrum of availability of amino acids in food, and the partitioning of protein expression intra- and extracellularly, guided pathway loss to converge on those nine [7]. However, differences in morphology, environment, and lifeways across these species are hard to reconcile with such a consistent pattern of amino acid synthesis loss.

## Perspective on recent results

### A new clue to their origin: EAAs and CEAAs have distinct codon profiles in the genetic code

**Figure 1:**
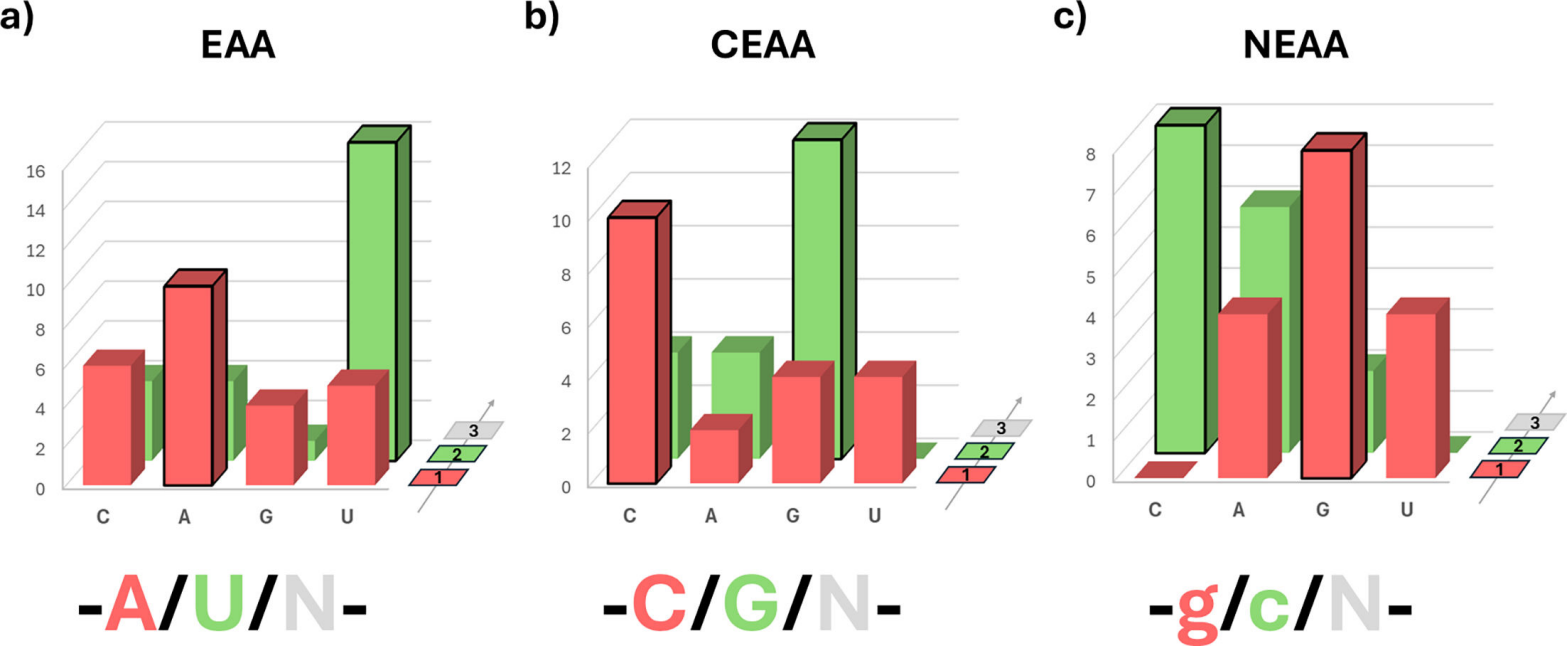
Distinct frequencies of nucleotide bases in position 1 (red) and position 2 (green) in codons are observed for the three nutritional classes of amino acids. The first two positions of a codon are the major information content carriers through which the corresponding amino acid is defined – the third, ‘degenerate’, position is a consequence of 64 possible codons being excessive when encoding just 20 amino acids and 3 stops. Moving from the first codon position (front, red) to the second (behind, green), the sum of instances of each nucleotide is displayed for each of the three nutritional groups, EAA (**a**), CEAA (**b**), and NEAA (**c**). Each group has a distinct nucleotide profile, and the resulting ‘modal’ codon is displayed underneath, with N representing any base at the third position. The lowercase nucleotides for (**c**) indicate their more indistinct nature.

It was recently noted that the three-way nutritional categorisation of amino acids is paralleled by a corresponding division of codon composition within the universal DNA genetic code [8]. A representation of nucleotide abundance at the first two codon positions for each nutritional amino acid grouping is shown below (Figure 1).

**Figure 2:**
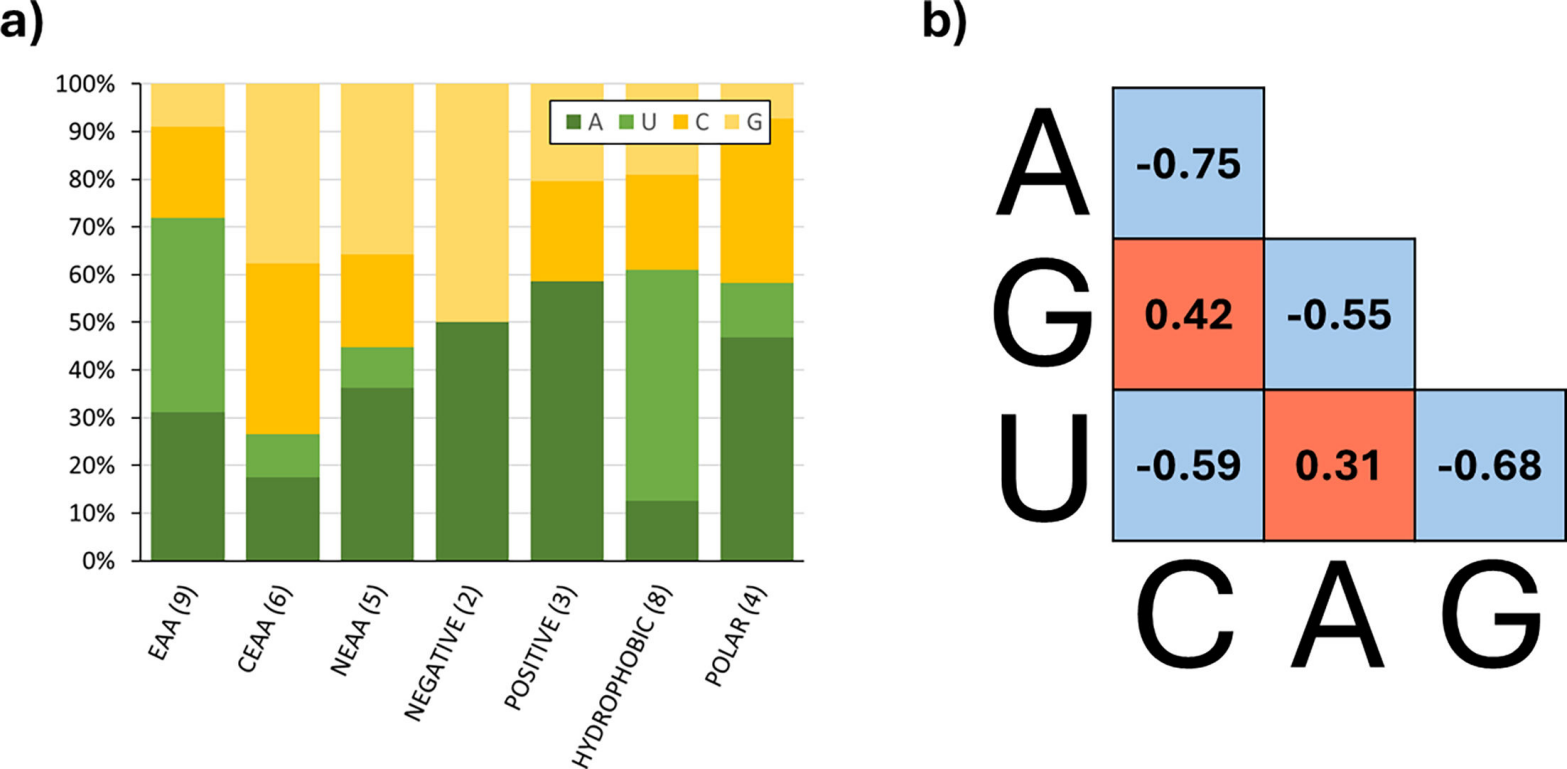
Biases in A/U and C/G nucleotide frequency are observed in the genetic code of nutritional amino acid groupings and in the human mRNA transcript population. In (**a**), the percentage composition of each of the four bases in the first two codon positions for each amino acid grouping has been calculated. Codon frequencies are not all equally common in humans, and so a correction factor has been used to take that into account. A and U nucleotides are far more common in EAA codons, whereas C and G nucleotides dominate CEAA codons. This ensures that the percentage figure represents the true demand for nucleotides for each grouping. In (b), the r^2^ correlations for the co-occurrence of all pairwise combinations of nucleotides within human transcripts (protein-coding component of the GENCODE v44) are displayed. Only C-G and A-U correlations are positive in direction (highlighted in red). All others show a negative correlation (blue). Thus, the natural trends in the compositional profiles of mRNA align with the nutritional amino acid groupings in the proteins they encode.

Characteristic nucleobase frequency biases are observed for the nutritional amino acid groupings. The resulting modal codons, **A-U-N** (for EAA) and **C-G-N** (for CEAA), are particularly striking. U at the second codon position is only ever observed in the encoding of EAAs. Below, Figure 2a presents unordered A/U and C/G paired nucleotide percentage frequencies within the first two codon positions (adjusted for human codon usage) across the three nutritional groupings and, for comparison, the standard chemical property-based groupings of amino acids. The extreme, and opposing, nucleotide frequency bias is uniquely a property of the EAA and CEAA amino acid groupings.

How might this observation have any bearing on the (potentially repeated) loss of EAA biosynthetic pathways? The existence of a safety net against the consequences of EAA starvation was proposed: a means by which the cell could selectively reduce the synthesis of messenger RNAs (mRNAs) encoding the EAA-rich proteins that may not be effectively translated during periods of dietary insufficiency [8]. This would economise EAA expenditure and avoid the cytotoxic effects of translation failure [9]. To achieve this requires that those mRNAs containing an excess of EAA-encoding codons become recognisable and repressed in some way. A novel form of mRNA synthesis regulation is required to achieve this.

### Hypothesis 1: nucleotide ‘supply-and-demand’ effects on mRNA expression may shield organisms against harmful consequences of EAA scarcity

The transcription of mRNA polymerises the building block nucleotide bases cytosine, adenine, guanine, and uracil (C/A/G/U). The promoter activity of each gene is the established determinant of transcription rate, defining levels of its corresponding mRNA (expression), and is thus a major factor in the expenditure of nucleotides. It was hypothesised that reduced supply of nucleotides might inhibit mRNA synthesis and expression. This was seen to be the case – and at a global scale, because any shortage affects the totality of transcription [8]. However, the relative frequencies of the four nucleotides within any mRNA transcript had a specific influence on its expression level in response to nucleotide shortage. In other words, the sequence of that mRNA represents the ‘demand’ in a supply-and-demand model of expression, with the availability of the nucleotides corresponding to the ‘supply’. Description of the demand aspect of expression control can be further simplified from four individual bases to pairs of nucleotides: A with U and C with G. In Figure 2b, the frequencies of nucleotides within all human mRNAs were correlated – positive correlations (r^2^ values) were only observed between A and U frequencies, and between C and G frequencies – all other correlations were negative. Additionally, increased A and U frequencies were associated with longer mRNA transcripts, and C and G with shorter transcripts [8]. These pairings are reminiscent of the A = T and C ≡ G hydrogen-bonding pairs that generate the complementarity of the double-stranded DNA helix. However, mRNA is largely single-stranded, with functional secondary ‘hairpin’ or ‘stem-loop’ structures probably only partially contributing to the evolution of base composition. Chargaff et al. [10] determined that the nucleotide [A]:[T] and [C]:[G] concentration ratios in sea urchin DNA approached 1, reflecting the complementary base pairing in the double helix (unknown at that time). However, the [A]:[G] ratio of 1.83 and the [T]:[C] ratio of 1.80 indicate the different proportions of purine and pyrimidine nucleotides within DNA. This must be reflected in a similar bias in the available free nucleotide pools within the cell, requiring carefully co-ordinated nucleotide metabolism processes to meet the compositional needs of DNA synthesis. It makes sense that mRNA synthesis (demand), ultimately drawing from the same underlying purine/pyrimidine pools, should also tend towards the observed equivalent [A]:[U] and [C]:[G] nucleotide ratios to maximise synthetic efficiency.

To summarise these findings, changes in relative and absolute nucleotide availability (supply) will have different effects on the synthesis/expression of each mRNA depending on its A/U and C/G composition (demand). The sum of these mRNA synthesis efficiencies establishes the global transcriptional profile. This phenomenon is impactful. Each organ and cell line has a specific global profile. Disrupted global mRNA expression profiles have been identified in multiple disorders, including schizophrenia, Alzheimer’s disease, and rheumatoid arthritis; multiple viral infections; and after treatment with ~20% of all drugs/chemicals analysed [8]. Nucleotide supply is clearly a vital lever in the cell’s regulation of mRNA expression. That supply is intrinsically sensitive to the synthetic availability of amino acid precursors, aspartate (NEAA), glutamine (CEAA), and glycine (CEAA), and the metabolic state indicators, ATP and phosphoribosyl pyrophosphate. In this way, nucleotide availability reflects the wider metabolic state of the cell. Most importantly, this novel form of gene expression regulation, with its A/U and C/G directionality, aligns with the A/U-rich and C/G-rich allocation of codons to EAAs and CEAAs, respectively. Thus, a new type of connection between mRNA and protein synthesis exists, and the safety net hypothesised above becomes a possibility.

A context for the Great Genome Deletion event can now be proposed, whereby any instance of starvation led to metabolic deficiencies affecting A/U nucleobase synthesis, resulting in reduced expression of a subset of the most A/U-rich mRNAs. These encode proteins that were particularly enriched in 9 of the 20 amino acids. These nine could afford to lose their synthetic pathways because of this protection during starvation. Thus, the nine EAAs were defined as a consequence of the natural genetic code ‘fault-line’ interacting with a specific form of supply-and-demand transcriptional regulation. The model also provides an explanation for hypothetical instances of repeated, independent Great Deletions of the same nine EAA pathways during eukaryotic evolution, because the genetic code and its fault lines are universal. One final observation is that this hypothetical model of EAA origin simultaneously creates the CEAAs as a diametrically opposed grouping, with C/G codon enrichment, and subject to C/G nucleotide supply regulation. While it is easy to comprehend the underlying biological ‘motives’ for evolved EAA regulation, the CEAAs remain something of an enigma, as they sit on the nutritional fence between synthesis and essentiality.

### Protein expression is also regulated by amino acid supply and demand

We analysed proteomic data to search for the consequences of uncertain amino acid supply on protein expression [11]. The prediction was that any shortfall in the amino acid building blocks of proteins would negatively impact resulting protein production, or expression levels. As mentioned above, the economic concept of ‘supply and demand’ was also useful to consider here because it highlighted the dual factors at play: the requirement for an adequate supply of EAAs and CEAAs, and the sequence-derived compositional spectrum of demand in the proteins to be synthesised in every cell. Proteins enriched in EAAs, for example, would struggle to be synthesised in the event of EAA shortage, with a reduction in their expression as the outcome. The expression of proteins rich in NEAAs would, by contrast, be comparatively unaffected by such a constraint. Using experimental proteomic data originating from well-fed animals or cell lines grown in purposefully amino acid-rich media, it did not prove possible to detect expression effects on EAA-enriched proteins. However, the proteins most enriched in CEAAs were universally expressed at lower levels, and this proposed insufficiency effect was further exacerbated by rapid cell proliferation, as *de novo* protein synthesis demands outstripped the struggling CEAA supply. Finally, it is important to point out that the protein expression effects observed here might be direct, as we proposed [11], or a secondary consequence of the mRNA regulation mechanism described here, but published later.

### Millennia of restricted EAA and CEAA supply have shaped the amino acid composition of proteins

It might be expected that natural selection would result in a population of proteins with compositions tightly clustered around a mean, offering the least burden on a potentially limited amino acid supply. However, the compositional distribution is remarkably broad, with extreme composition proteins often observed [11]. Proteins extremely rich in CEAAs are over-represented in specific cellular processes and functionalities. Most obvious is the abundance of connective tissue, skin, nail, and hair proteins, such as collagens, elastin, keratin-associated proteins, late cornified envelope proteins, small cysteine-, glycine-, and proline-repeat-containing proteins, fibrillins, and fibulins. It seems likely that the permanent shedding of these tissues from the body has shaped their protein sequences to be very low in valuable EAA resources. Nevertheless, periods of rapid growth or illness will also put a strain on the expression of these CEAA-rich proteins, perhaps explaining the skin and hair phenotypes associated with chronic illness. The metabolic disorder, homocystinuria, features reduced levels of cysteine, a CEAA, and may be thought of as a genetic mimic of cysteine starvation. Historically, homocystinuria diagnosis was often confused with Marfan syndrome because they share pathologies of lens abnormalities and accelerated growth rates [12,13]. Loss-of-function mutations in the connective tissue gene, *FBN1*, underlie classic Marfan’s syndrome, and the corresponding protein, fibrillin-1, is notable for its very high CEAA composition. An explanation for the diagnostic confusion, proposed here, is that cysteine deficiency produces a phenocopy of Marfan syndrome by reducing FBN1 protein expression according to the supply-and-demand model. Defects in skin and hair are also observed in homocystinuria, further supporting a novel interpretation of this disease’s aetiopathology based on reduced expression of CEAA-rich proteins.

At the other end of the amino acid composition spectrum are those proteins with extreme EAA richness. There are many members of the taste and olfactory receptor superfamilies, and these are discussed in more detail later. Excluding them from analysis exposes the mitochondrial electron transport chain, glycosylation, fatty acid metabolism, protein trafficking, and chemokine signalling as processes that may be particularly negatively affected by EAA supply limitation. These are the protein families and functionalities that may be first affected by dietary insufficiency, and their down-regulation might be key to the survival of the organism by reducing metabolic expenditure.

### Hypothesis 2: the EAA composition of proteins has evolved to integrate with hunger and reproductive physiology

At the cellular level, amino acid insufficiency is detected by the mTORC1 and GCN2/eIF2/ATF4 signalling pathways, resulting in homeostatic transcriptional and translational adaptations, as well as the salvage of internal amino acids through autophagy [14]. Ultimately, however, it is an organism’s behavioural responses to amino acid shortage that dictate survival. Most immediate is the organism’s need to actively seek out food sources. The study of mouse and human obesity genetics has been instrumental in identifying the molecular signals connecting the nutritional state of peripheral tissues with the hypothalamic circuitry in the brain controlling hunger/satiety. These pathways drive the organism to seek out food when nutrients (particularly lipids) are reduced in bodily storage. Peripheral adipocyte secretion of leptin (LEP) hormone – proportionate to the degree of adiposity – signals a healthy nutritional status to the hypothalamus via its receptor (LEPR). Downstream signalling through melanocortin 4 receptor (MC4R)-expressing neurons converts the leptin signal into behavioural satiety. However, long-term starvation-induced reduction in adipocytes lowers leptin secretion, and the reduced MC4R activation triggers hunger and food-seeking behaviours. In addition to this long-term control of hunger, there are short-term controls. Insulin and glucagon work in concert to regulate blood glucose levels over the short term. Insulin and glucose levels can also be detected by the brain, producing a ‘glucostatic’ effect on hunger. Similarly, high levels of protein in a meal reduce concentrations of the hunger-promoting ghrelin hormone while also increasing peptide YY (PYY) hormone release, both decreasing feelings of hunger.

Like lipid storage in adipocytes, the EAA status of an organism needs to be part of a long-term system of dietary management. Extending the ideas presented earlier, if a protein regulator of hunger was enriched in EAAs, then this would limit its translational capacity during long-term insufficiency, reducing its expression and resulting in consequences to those hunger behaviours. The same mechanism could also be beneficial in reproductive regulator proteins because the decision to reproduce pits the need for species survival against the needs of (maternal) nutritional resource management and future resource availability for offspring after birth.

**Figure 3:**
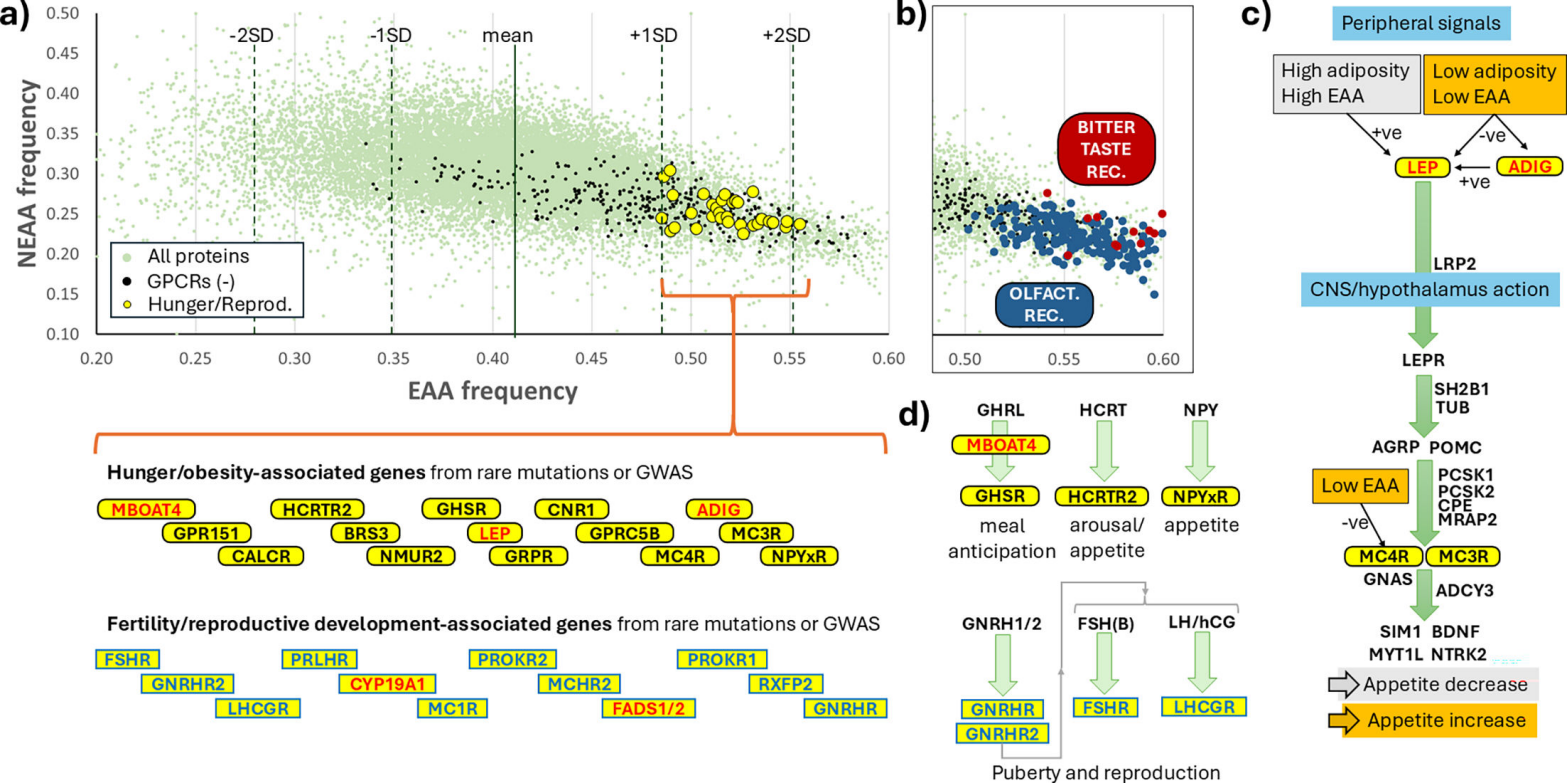
Key protein regulators of hunger and reproductive physiology have gained functional sensitivity to EAA nutritional state by evolving sequences enriched in EAA. In (**a**), almost 20,000 human proteins are shown on a scatterplot according to their EAA and NEAA compositions (green points). Proteins linked to hunger/reproduction pathways by genetic studies, and with EAA compositions greater than one standard deviation above the mean, are shown superimposed (yellow circles). Their protein symbols are listed below the plot as yellow rectangles (reproduction) or yellow ovals (hunger), and positioned according to increasing relative EAA richness left-to-right. Black text indicates GPCR proteins, whereas red text highlights non-GPCR proteins. For example, the pivotal reproductive GPCR protein, GNRHR, is ranked 161st most EAA-rich out of 19,943 proteins (after exclusion of the olfactory and bitter taste receptor clusters). This places it two standard deviations above the mean. All GPCRs, except for the large bitter taste and olfactory subfamilies, have also been plotted as black points to illustrate their inherent compositional bias towards EAA richness. In (b), the extreme EAA compositions of the olfactory and bitter taste receptors are highlighted. In (c), the leptin–melanocortin pathway of hunger regulation is shown to contain four proteins, LEP, ADIG, MC3R, and MC4R, with an extreme EAA composition. The pathway triggers shown in grey rectangles increase leptin expression and, thus, decrease appetite, whereas insufficiencies in both lipids and EAAs (orange rectangles) result in reduced leptin expression and pathway response, leading to an increase in hunger and food-seeking behaviours. In (d), further hunger, pubertal development, and fertility pathways illustrate the high EAA composition, and potential sensitivity, of their receptor components. Abbreviations: GNRHR, gonadotropin-releasing factor receptor; MC3R, melanocortin 3 receptor.

Figure 3a adapts ideas first presented in [11], showing all human proteins plotted according to EAA and NEAA composition. Genetic studies of hunger, obesity, reproduction, and fertility were used to identify major contributory genes, and the subset encoding proteins with an elevated EAA composition was identified [15–26]. The majority of these were G protein-coupled receptors (GPCRs). The GPCR superfamily tends to have an above-average proportion of EAAs, which may simply reflect their sequence homology and functional constraint but, alternatively, may indicate that GPCR sequences have evolved so that EAA shortfall acts as a systems-wide means to regulate sensory and endocrine signalling. In Figure 3b, the GPCRs of the olfactory and bitter taste (TAS2Rx) families are shown as particularly extreme EAA composition outliers. The well-documented reduction in bitter taste sensation during starvation [27,28] could be explained by constrained receptor expression as EAA availability drops. This may serve an evolutionary advantage by opening up a greater range of food sources that would not all be considered palatable during protein diet sufficiency, albeit at greater risk of exposure to toxicity or infection. An equivalent reduction in olfactory sensation has not been recorded during starvation – in fact, the consensus is that smell perception becomes more acute during hunger [29,30] and is only dulled as a consequence of obesity [31]. However, this is not a universal effect for all odour detection, and there is evidence that the enhancement of smell may have an electrophysiological component [30].

The most striking observation is shown in Figure 3c: leptin (LEP) hormone, its adipocyte regulator, adipogenin (ADIG) [32], and downstream effector MC4R are all highly enriched in EAAs. Importantly, the hypothetical reduction in expression of all these proteins during EAA shortage would additively increase feelings of hunger, mimicking the effects of reduced adiposity. Thus, this major pathway of lipid hunger control may be co-regulated by EAA levels. This has obvious consequences for adherence to present-day weight-loss strategies – maintaining protein intake would be important as net caloric input and body mass index (BMI) decrease. In Figure 3d, other hunger-controlling signals, such as ghrelin (GHRL), hypocretin (‘orexin’, HCRT), and neuropeptide Y (NPY), possess regulators and receptors enriched in EAAs.

The effects of leptin and melanocortin receptors on appetite regulation spill over into the regulation of reproductive development and ongoing fertility [33–35]. Within the reproductive pathways themselves, key pubertal development and fertility factors, such as gonadotropin-releasing hormone and its downstream effectors, follicle-stimulating hormone (FSH/B) and luteinising hormone, signal through GPCRs that are all highly enriched in EAAs. Not all proteins affecting reproductive pathways are GPCRs. CYP19A1 (‘aromatase’ enzyme) is a key fertility protein, converting androgens into oestrogens, but it also regulates hunger pathways [36]. The fatty acid metabolism enzymes FADS1 and FADS2 have been linked to fertility/reproductive success and are the focus of strong evolutionary selection as human diets changed in the period after the prehistoric introduction of agriculture/domestication [25,37].

## Conclusion

The regulation by substrate concentration of two key polymerisation processes in the cell – transcription and translation – adds a new perspective on the control of expression by facilitating a closer relationship with the general metabolic state of the cell and the organism. This, in turn, has permitted evolution to link EAA availability to survival pathways by selection pressure on protein sequence composition. Thus, the evidence points to the presence of EAAs as a help, rather than a hindrance, for animal kingdom domination of the Earth.

The hypotheses presented here require testing. While linking EAA emergence to the genetic code is conveniently difficult to prove one way or the other, converting *in silico* observations on EAA availability and expression level into *in vitro* and *in vivo* experiments is readily achievable and a vital next step. If this mechanism proves to be an important contributor to expression regulation, it will add clarity and opportunity to many areas where dietary impoverishment or excess exists – from malnutrition and obesity, through to cancer and the treatment of infertility.

## Abbreviations

CEAA: conditionally essential amino acid
EAA: essential amino acid
GPCR: G protein-coupled receptor
MC4R: melanocortin 4 receptor
NEAA: non-essential amino acid
mRNA: messenger RNA
